# LncRNA DUXAP8 induces breast cancer radioresistance by modulating the PI3K/AKT/mTOR pathway and the EZH2-E-cadherin/RHOB pathway

**DOI:** 10.1080/15384047.2022.2132008

**Published:** 2022-11-03

**Authors:** Changjiang Lei, Shaoting Li, Ying Fan, Li Hua, Qingyun Pan, Yuan Li, Zhixiong Long, Rui Yang

**Affiliations:** a Department of General Surgery, the Fifth Hospital of Wuhan, Wuhan, China; b Department of Pharmacy, the Fifth Hospital of Wuhan, Wuhan, China; c Department of Cardiology, the Fifth Hospital of Wuhan, Wuhan, China; d Department of Medical Examination Center, the Fifth Hospital of Wuhan, Wuhan, China; e Department of Blood Endocrinology, the Fifth Hospital of Wuhan, Wuhan, China; f Department of Oncology, the Fifth Hospital of Wuhan, Wuhan, China

**Keywords:** LncRNA DUXAP8, AKT, EZH2, breast cancer, radioresistance

## Abstract

Radiation resistance poses a major clinical challenge in breast cancer (BC) treatment, but little is known about how long noncoding RNA (lncRNA) may regulate this phenomenon. Here, we reported that DUXAP8 was highly expressed in radioresistant BC tissues, and high expression of DUXAP8 was associated with poor prognosis. We found that the overexpression of DUXAP8 promoted radioresistance, while the knockdown of DUXAP8 expression increased radiosensitivity. Further studies revealed that DUXAP8 enhanced the radioresistance of BC cells by activating the PI3K/AKT/mTOR pathway and by repressing the expression of E-cadherin and RHOB through interaction with EZH2. Together, our work demonstrates that the overexpression of DUXAP8 promotes the resistance of BC cells toward radiation through modulating PI3K/AKT/mTOR pathway and EZH2-E-cadherin/RHOB axis. Targeting DUXAP8 may serve as a potential strategy to overcome radioresistance in BC treatment.

## Background

Breast cancer (BC) is the most frequent cancer among women, with an estimated 1.5 million new cases per year.^1,2^ Radiation therapy plays an important role in the multidisciplinary management of BC.^3^ However, the intrinsic and acquired radioresistance remains a major challenge undermining the treatment outcome of radiotherapy in BC patients.^4^ Therefore, the exploration and clarification of molecular mechanisms implicated in the development of BC radioresistance would provide insights into the formulation of novel strategy in the management of radiotherapy resistance.

Non-coding RNAs (ncRNAs), which can be classified into long ncRNAs (lncRNAs, ˃ 200 nt) and small ncRNAs (˂ 200 nt), have emerged as critical regulators of gene expression.^5^ As one major class of small ncRNAs, microRNAs (miRNAs) regulate the stability and translation of target mRNA by binding to the 3′-untranslated region.^6^ Previous studies have shown that miRNAs regulate a myriad of cellular and developmental processes, including the initiation and progression of cancer.^6^ Recent reports further showed that many lncRNAs are aberrantly expressed in human tumor tissues, which contributes to the malignant phenotypes of cancers, including the hyper-proliferation, cell invasion, metastasis and radioresistance.^5,7–9^ For instance, lncRNA NEAT1 acts as an oncogenic factor in various cancers, and functions as competitive endogenous RNAs to sponge downstream miRNAs.^5,7–9^ Certain lncRNAs could serve as bridge to target polycomb factor EZH2 (Enhancer Of Zeste 2 Polycomb Repressive Complex 2 Subunit) to its target gene loci, causing epigenetic silencing of tumor suppressors.^5–10^ In addition to ncRNAs, the dysregulation of many signaling pathways play crucial roles in the progression of BC.^11^ For example, the constitutive activation of PI3K/AKT/mTOR pathway confers a competitive growth advantage, metastatic competence and drug resistance in BC cells.^11^

LncRNA DUXAP8 is located on chromosome 20q11, and the upregulation of DUXAP8 has been reported in multiple cancers, including gastric cancer,^12^ lung cancer,^13^ bladder cancer,^14^ renal cell carcinoma,^15^ hepatocellular carcinoma,^16^ neuroblastoma^17^ and colorectal cancer.^18^ DUXAP8 acts as an oncogene in several cancer types through an EZH2-dependent mechanism^13^ or by regulating PTEN expression.^14^ However, the expression pattern and impact of DUXAP8 on development of BC radioresistance remain unknown.

In this study, we aimed to figure out whether DUXAP8 modulates the radiosensitivity of BC cells and investigate the underlying mechanisms of DUXAP8-dependent radioresistant phenotype. We found that the overexpression of DUXAP8 could enhance the radioresistance of BC cells through activating PI3K/AKT/mTOR pathway and repressing the expression of EZH2 target genes (E-cadherin and RHOB). These data indicate that targeting DUXAP8 might serve as a therapeutic approach for the management of the radiotherapy efficacy in BC.

## Materials and methods

### Patients and tissue specimens

In this study, BC specimens and the matched noncancerous tissues were obtained from 50 patients who were diagnosed with BC and underwent surgery at the Fifth Hospital of Wuhan (China). All the cancer tissues and adjacent normal tissues were confirmed by experienced pathologists. No patient received any chemotherapy or radiotherapy before surgery. Tissue samples were immediately frozen in liquid nitrogen after surgical resection and stored at −80°C until use. A total of 60 BC patients who received radiotherapy after surgery were also enrolled in the study. These tissue specimens were divided into radiosensitive group (n = 30) and radioresistant group (n = 30) based on short-term response to radiotherapy, as previously described.^19^ Clinicopathological data were retrieved from patient medical records. This study was approved by the institutional ethical review committee of the Fifth Hospital of Wuhan (China), and written informed consent was obtained from each patient.

### Cell lines and culture

Human BC cell lines, including MCF-12A, MCF-12 F, MCF-7, T47D, ZR-75-1, HCC-1806, MDA-MB-468, BT-549, and MDA-MB-231, and the normal mammary epithelial cell line MCF-10A were obtained from the Cell Bank of Type Culture Collection (Chinese Academy of Sciences, Shanghai, China). The cells were maintained in DMEM/F12 medium (Thermo Fisher Scientific, USA) supplemented with 10% fetal bovine serum (Thermo Fisher Scientific, USA) in a humidified incubator containing 5% CO_2_. To study the impact of indicated signaling pathways, cells were treated with target-selective inhibitor of PI3K, NVP-BKM120 (4 µM, Novartis, Basel, Switzerland) or DMSO (Sigma, St. Louis, USA) for 24 h before subsequent experiments.

### Quantitative real-time PCR (qRT-PCR)

Total RNA was extracted from cultured cells or tissues using the TRIzol Reagent (Invitrogen, USA), and then reverse-transcribed into cDNA with the PrimerScript RT Reagent Kit (Invitrogen, USA). qRT-PCR analysis was performed using the SYBR Green PCR kit from Takara Biotechnology (Takara, Dalian, China) in a 7500 Real Time PCR System (Applied Biosystems, CA, USA). GAPDH was used as an internal control. Primers used for qRT-PCR assay were obtained from GenePharma (China, Shanghai) and shown as follows: human DUXAP8, forward: 5′-ACCAGCCTCACTAGCACTCT-3′ and reverse: 5′-GGCTTAGCTTGCACTTTTGGA-3′; human EZH2, forward: 5′-AATCAGAGTACATGCGACTGAGA-3′ and reverse: 5′-GCTGTATCCTTCGCTGTTTCC-3′; human p21, forward: 5′-TGTCCGTCAGAACCCATGC-3′ and reverse: 5′-AAAGTCGAAGTTCCATCGCTC-3′; human Bax, forward: 5′-CCCGAGAGGTCTTTTTCCGAG-3′ and reverse: 5′-CCAGCCCATGATGGTTCTGAT-3′; human Caspase-8, forward: 5′-TTTCTGCCTACAGGGTCATGC-3′ and reverse: 5′-GCTGCTTCTCTCTTTGCTGAA-3′; human Caspase-9, forward: 5′-CTTCGTTTCTGCGAACTAACAGG-3′ and reverse: 5′-GCACCACTGGGGTAAGGTTT-3′; human PTEN, forward: 5′-TGGATTCGACTTAGACTTGACCT-3′ and reverse: 5′-GGTGGGTTATGGTCTTCAAAAGG-3′; human E-cadherin, forward: 5′-CGAGAGCTACACGTTCACGG-3′ and reverse: 5′-GGGTGTCGAGGGAAAAATAGG-3′, human RHOB, forward: 5′-CTGCTGATCGTGTTCAGTAAGG-3′ and reverse: 5′-TCAATGTCGGCCACATAGTTC-3′; and human GAPDH, forward: 5′-GGAGCGAGATCCCTCCAAAAT-3′ and reverse: 5′-GGCTGTTGTCATACTTCTCATGG-3′.

### Plasmid construction and cell transfection

The full-length cDNA sequence of human DUXAP8 was amplified by PCR using forward primer: 5’-GCGTGGTCAGAGCGAGCTT-3’; reverse primer: 5’-GCTTAGCTTGCACTTTTGGAAGA-3’. The resulting PCR fragment was cloned into the pcDNA3.1 vector (Invitrogen, USA). Stable BC cell lines overexpressing DUXAP8 were generated by the transfection of DUXAP8 expression vector or the control pcDNA3.1 vector into MCF-7 and T47D cells with low DUXAP8 expression. Stable BC cell lines with DUXAP8 silencing were generated by the transfection of DUXAP8-specific shRNA vector or the control shRNA vector (GenePharma, Shanghai, China) into BT-549 and MDA-MB-231 cells with high DUXAP8 expression. Transfection was performed using Lipofectamine 3000 (Invitrogen, L3000001) according to the manufacturer’s instructions. Transfected cells were selected with 1.0 μg/mL puromycin for two weeks to eliminate the uninfected cells as previously described.^20^

### Oligonucleotides and siRNAs transfection

Small interfering RNAs (siRNAs) against human EZH2, E-cadherin and RHOB, and scrambled siRNA were purchased from GenePharma (China, Shanghai). BC cells were transfected with 50 nm of above siRNAs using Lipofectamine 2000 (Invitrogen, USA) following the manufacturer’s guides.

### Cell viability assay

To determine cell viability, cells were seeded in to a 96-well plate at a density of 5000 cell/well and cultured in a humidified cell culture incubator for 12 h. BC cells were exposed to the indicated radiation doses, and the cell viability was determined using a CCK-8 kit (Dojindo Laboratories, Kumamoto, Japan) according to the manufacturer’s instructions. The absorbance was determined at 450 nm using a microplate reader (Bio-Rad Laboratories, USA).

### Cell apoptosis detection

BC cells seeded into 6-well plates and incubated for 24 h, and then were irradiated with 0 to 8 Gy for 24 h. For apoptosis detection, BC cells were first digested by trypsin and re-suspended with binding buffer, and the staining for apoptotic cells was detected using an Annexin V-FITC Apoptosis Detection Kit (BD Biosciences, USA) as previously reported.^19^ The percentage of apoptotic cells was detected by BD FACS CantoTM II Flow Cytometer (BD Biosciences), and the data were analyzed using CellQuest software (BD Biosciences, USA).

### Western blotting analysis

The total protein of cells was extracted using RIPA lysis buffer (Solarbio, Shanghai, China). The protein concentration was quantified using the Pierce BCA protein assay kit (Thermo Fisher Scientific, USA). The protein extracts were subjected to SDS-PAGE, and then transferred to 0.22 μm polyvinylidene difluoride (PVDF) membrane (Millipore, USA). After blocking with 5% nonfat milk, the membranes were incubated at 4°C overnight with primary antibodies against γ-H2AX, cleaved caspase-7/8/9, Bax, PTEN, p-PI3K, PI3K, p-AKT, AKT, p-mTOR, mTOR, EZH2, E-cadherin, RHOB, Ki-67, and GAPDH (all antibodies from Cell Signaling Technology, USA, at 1:1000 dilutions). The membrane was then incubated with HRP-linked secondary antibody (1:3000; Cell Signaling Technology) at room temperature for 2 h. Signals were developed using an ECL Western Blotting Detection Kit from GE Healthcare (Amersham, UK). The protein bands were photographed on a gel imager system (Bio-Rad, CA, USA). The densitometry analysis was performed with Image J software (Bethesda, MD, USA).

### Subcellular fractionation

The separation of nuclear and cytoplasmic fractions was performed using the PARIS Kit (Thermo Fisher Scientific, USA) according to the manufacturer’s instructions. The qRT-PCR analysis was performed to quantify the relative abundance of the target using the separated nuclear and cytoplasmic fractions. U6 served as the nuclear control, and GAPDH served as the cytoplasmic control.

### RNA immunoprecipitation (RIP)

RIP experiments were conducted according to the manufacturer’s protocol of the Magna RIP RNA-Binding Protein Immunoprecipitation Kit (Millipore, USA). In brief, magnetic beads were pre-incubated with anti-EZH2 or anti-rabbit IgG antibodies (Cell Signaling Technology) for 30 min, and the cell lysates were immunoprecipitated with beads overnight at 4°C with rotation. RNA was purified from RNA-protein complexes bounded to the beads using TRIzol reagent and was analyzed by qRT-PCR analysis.

### Chromatin immunoprecipitation assay (ChIP)

ChIP experiments were performed using the Millipore EZ-Magna ChIP Kit (Millipore, USA) as previously reported.^21^ Briefly, BC cells were cross-linked with 1% formaldehyde for 10 min at room temperature. Then, chromatin was sonicated in the lysis buffer and the extraction of ChIP DNA was performed according to the kit’s protocol. The antibodies for ChIP were EZH2 and H3K27Me3 (Cell Signaling Technology, USA). The primer sequences for ChIP-qPCR analysis were previously described ^2223^.

### In vivo experiments

The experiments involving animals were approved by the Animal Care and Use Committee of the Fifth Hospital of Wuhan and were performed in accordance with the Institutional Guide for the Care and Use of Laboratory Animals. Six-week-old nude mice were purchased from Shanghai Laboratory Animal Center, Chinese Academy of Sciences (Shanghai, China), and housed in specific pathogen-free conditions on a 12-h light/dark cycle with free access to food and water. 0.2 mL of cell suspension containing 1 × 10^6^ cells were implanted into nude mice by subcutaneous injection (n = 6 mice in each group). Two weeks after injection, mice were irradiated with 8 Gy as previously reported.^19^ Tumor sizes were measured every 5 days for 7 weeks, and tumor volume was calculated according to the following formula: Volume = (length x width^2^)/2. Seven weeks after tumor cell inoculation, all the mice were euthanized by CO_2_ asphyxiation, and the death was confirmed by t cervical dislocation. The xenograft tumors of terminally dead mice were removed for subsequent analysis.

### Statistical analysis

All data were expressed as mean ± SD and were analyzed using SPSS 17.0 (SPSS Inc, USA). The differences were estimated using Student’s *t*-test (between two groups) or one-way ANOVA (among at least three groups). Fisher’s exact and chi-square tests were utilized to evaluate the correlations between DUXAP8 expression level and clinical parameter in BC patients. The correlation between DUXAP8 and EZH2 expression in BC tissues was determined by Pearson correlation analysis. The results were considered statistically significant if *P* < .05.

## Results

### DUXAP8 is up-regulated in radioresistant BC tissues and high DUXAP8 expression predicts poor clinical outcomes in BC patients

**Figure 1.**
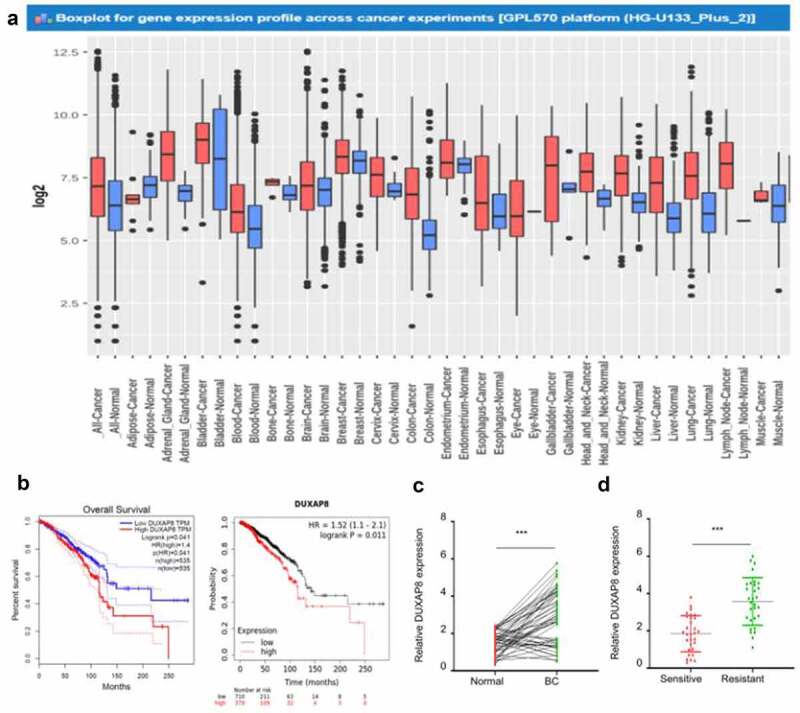
DUXAP8 is up-regulated in radioresistant BC tissues and high DUXAP8 expression predicts poor prognosis in BC patients.

To understand the biological role of DUXAP8 in BC, we first investigated whether DUXAP8 was dysregulated in BC tissues. Using the GENT2 database (http://gent2.appex.kr/gent2/), we found that the expression level of DUXAP8 in BC tissues was much higher than that in normal tissues (*P* = .001, Figure 1a). We further employed the GEPIA database (http://gepia.cancer-pku.cn/) and the Kaplan Meier Curve plotter database (http://kmplot.com) to analyze the relationship between DUXAP8 level and the prognosis of BC patients. Kaplan-Meier survival analysis suggested that BC patients with higher levels of DUXAP8 were associated with an poorer overall survival compared with those with a lower level of DUXAP8 (Figure 1b). Subsequently, we examined the levels of DUXAP8 in 60 paired BC and adjacent normal breast tissues using qRT-CR analysis. The results also showed a significant upregulation of DUXAP8 in BC specimens compared with adjacent normal tissues (*P* < .0001; Figure 1c). Analysis of the relationship between DUXAP8 expression and clinicopathological characteristics (Table 1) revealed that elevated DUXAP8 expression was significantly associated with larger tumor size, more advanced tumor stage, and more lymph node metastasis. These results suggested that the upregulation of DUXAP8 may contribute to the BC progression.

**Table 1.** Analysis of the relationship between DUXAP8 expression level and clinicopathological characteristics of breast cancer patients.

Factor		DUXAP8 expression		*P* value
		Low (n = 30)	High (n = 30)	
Age				0.301
	≤65	14	18	
	>65	16	12	
Tumor size				0.037
	≤3 cm	17	9	
	>3 cm	13	21	
Tumor differentiation				0.02
	Well/moderate	21	12	
	Poor	9	18	
TNM stage				0.02
	I/II	19	10	
	III/IV	11	20	
Lymph node metastasis				0.037
	Negative	21	13	
	Positive	9	17

(**a**) The mRNA levels of DUXAP8 in different types of tumors in the GENT2 database. (**b**) Probability of overall survival in BC patients expressing high or low DUXAP8 levels assessed using GEPIA database (left) and KM plotter (right) databases. (**c**) qRT-CR analysis of DUXAP8 levels in 50 paired BC and adjacent normal breast tissues. (**d**) qRT-CR analysis of DUXAP8 expression in radiosensitive BC patients (n = 30) and BC radioresistant patients (n = 30). ***P < .001.

To explore whether the dysregulation of DUXAP8 is correlated with radiosensitivity in BC patients, we analyzed the expression of DUXAP8 in the BC tissues from radiosensitive and radioresistant patients using qRT-PCR analysis. Our results demonstrated that the expression levels of DUXAP8 were remarkably higher in the radioresistant group than those in the radiosensitive group (Figure 1d). The median level of DUXAP8 from qRT-PCR experiments was used the cutoff point to divide BC patients into high- (n = 30) and low- (n = 30) expression groups. Compared with DUXAP8-low expression group, the DUXAP8-high expression group exhibited a poorer short-term response to radiotherapy (Table 2). Overall, these results suggest that DUXAP8 might act as an oncogenic factor contributing to the BC progression and conferring resistance to radiation therapy in BC cells.

**Table 2.** Comparison of tumor responses to radiotherapy between the DUXAP8-low expression group and the DUXAP8-high expression group.

Tumor response (n)	DUXAP8-low expression (n = 30)	DUXAP8-high expression (n = 30)	*P*-value
Complete response (CR)	2	1	0.028
Partial response (PR)	5	2	
Stable disease (SD)	11	8	
Progressive disease (PD)	12	19	

### Overexpression of DUXAP8 enhances the radioresistance of BC cells by activating the PI3K/AKT/mTOR pathway

**Figure 2.**
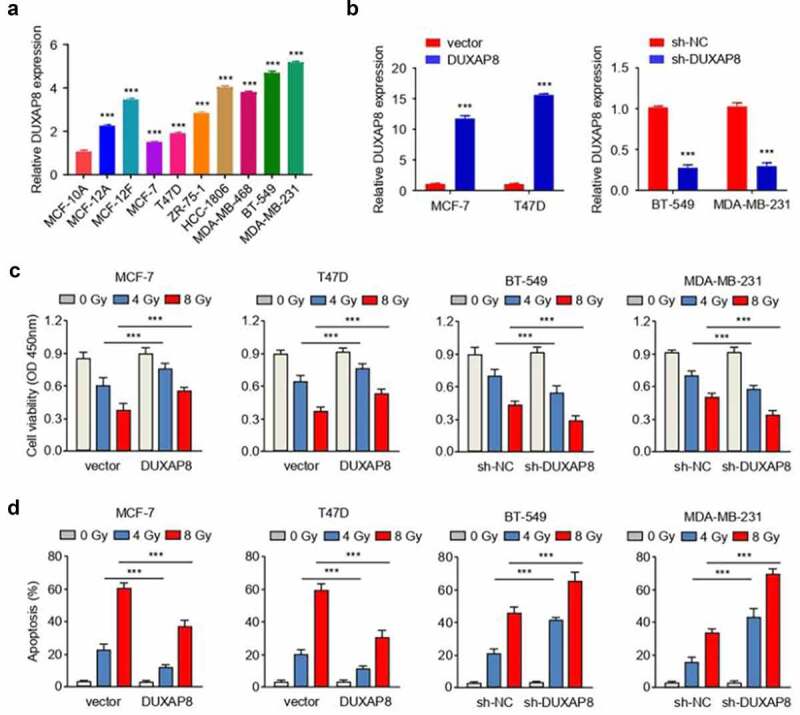
DUXAP8 promotes the radioresistance of BC cells.

We next determined the expression levels of DUXAP8 in normal breast epithelial cell MCF-10A and several BC cell lines (MCF-12A, MCF-12 F, MCF-7, T47D, ZR-75-1, HCC-1806, MDA-MB-468; BT-549 and MDA-MB-231) by qRT-PCR assays. Compared to MCF-10A cells, BC cells showed a significant increase in the expression of DUXAP8 (Figure 2a). BT-549 and MDA-MB-231 cells expressed the highest levels of DUXAP8, while MCF-7 and T47D cells showed the lowest expression (Figure 2a). Thus, we selected MCF-7 and T47D with low DUXAP8 expression for overexpression experiments, and BT-549 and MDA-MB-231 cells with high DUXAP8 expression for knockdown experiments.

(**a**) qRT-PCR analysis of DUXAP8 expression in the normal breast epithelial cell MCF-10A and BC cell lines (MCF-12A, MCF-12 F, MCF-7, T47D, ZR-75-1, HCC-1806, MDA-MB-468; BT-549 and MDA-MB-231). (**b**) qRT-PCR analysis of DUXAP8 expression in MCF-7 and T47D cells with DUXAP8 overexpression and in BT-549 and MDA-MB-231 cells with DUXAP8 knockdown. (**c, d**) CCK-8 viability assays (**c**) and flow cytometry analysis (**d**) showed that the overexpression of DUXAP8 promoted cell survival, and reduced the apoptosis of MCF-7 and T47D cells after radiation treatment (0, 8 Gy). Conversely, the knockdown of DUXAP8 impaired cell survival and promoted apoptosis of BT-549 and MDA-MB-231 cells after radiation treatment. ***P < .001.

**Figure 3.**
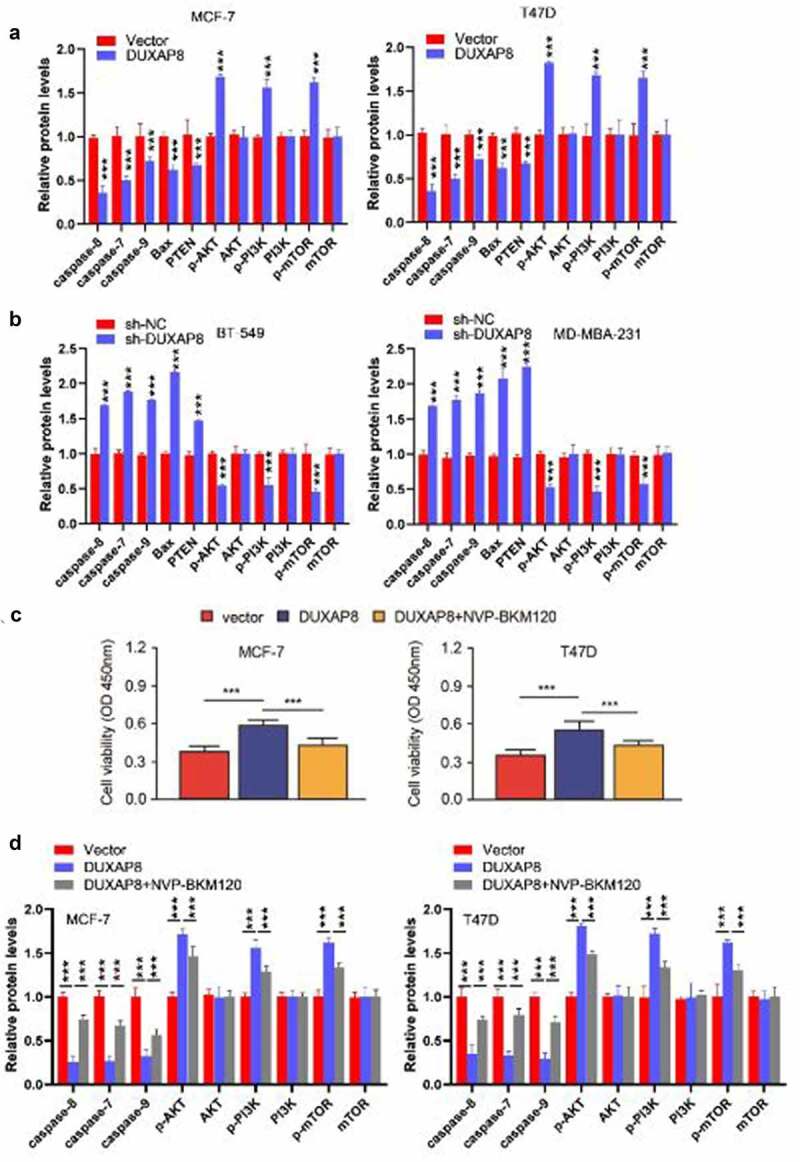
DUXAP8 enhances the radioresistance of BC cells by activating the PI3K/AKT/mTOR pathway.

To confirm the role of DUXAP8 in radiation resistance in BC cells, DUXAP8 was over-expressed by transfecting BC cells with DUXAP8 expression vector, or knocked down by transfecting BC cells with shRNA targeting DUXAP8 (Figure 2b). The cells were subjected to different dosage of radiation and CCK-8 viability assays and flow cytometry analysis were performed to examine cell viability and apoptosis. We found that the overexpression of DUXAP8 promoted cell survival and reduced apoptosis of MCF-7 and T47D cells after radiation treatment (0, 4, 8 Gy) (Figure 2 c and D). Conversely, the knockdown of DUXAP8 impaired cell survival and promoted the apoptosis of BT-549 and MDA-MB-231 cells after radiation treatment (Figure 2 c and d**; Figure S1**). Consistently, Western blot analysis showed that overexpression of DUXAP8 significantly decreased γ-H2AX (a marker for DNA damage) levels after irradiation, while the knockdown of DUXAP8 showed the opposite effects (**Figure S2**), which further indicates that overexpression of DUXAP8 reduces the level of DNA damage upon radiation in BC cells. Moreover, the overexpression of DUXAP8 led to a significant reduction in PTEN (negative regulator of PI3K signaling pathway), Bax (pro-apoptotic protein), and cleaved caspase-8/7/9 (apoptotic executors), while there were significant reductions in the phosphorylation levels of PI3K, AKT and mTOR. The knockdown of DUXAP8 showed the opposite effects (Figure 3 a and b**; Figure S3, upper panel**). Together, these data suggest that DUXAP8 mediate the radiosensitivity by modulating PI3K/AKT/mTOR pathway.

(**a, b**) Protein levels of PTEN, Bax, cleaved caspase-8/7/9, p-PI3K, PI3K, p-AKT, AKT, p-mTOR and mTOR were determined by Western blot in MCF-7 and T47D cells with DUXAP8 overexpression (**a**), and in BT-549 and MDA-MB-231 cells after DUXAP8 knockdown (**b**). (**c**) MCF-7/T47D cells overexpressing DUXAP8 or the respective control cells were treated with a selective inhibitor of PI3K (NVP-BKM120). Then, these cells were exposed to different doses of radiation and cell viability was measured using CCK-8 assays. (**d**) Protein levels of p-PI3K, PI3K, p-AKT, AKT, p-mTOR and mTOR, and cleaved caspase-8/7/9 levels in MCF-7 and T47D cells shown in (**c**). ***P < .001.

To further confirm whether DUXAP8 mediates the radioresistance of BC cells by activating the PI3K/AKT/mTOR pathway, MCF-7/T47D cells overexpressing DUXAP8 were treated with a selective inhibitor of PI3K (NVP-BKM120), and the cell viability upon irradiation was examined determined by CCK-8 assay. As expected, overexpression of DUXAP8 promoted the survival of MCF-7 and T47D cells after the treatment of 8 Gy IR (Figure 3c). However, the treatment of PI3K inhibitor NVP-BKM120 significantly impaired the effects of DUXAP8 overexpression on cell viability (Figure 3c). Western blot analysis showed that the overexpression of DUXAP8 increased the phosphorylation levels of PI3K, AKT and mTOR, and decreased the levels of cleaved caspase-8/7/9 in MCF-7 and T47D cells (Figure 3d**; Figure S3, bottom panel**). However, the treatment of PI3K inhibitor significantly decreased the phosphorylation levels of PI3K, AKT and mTOR and increased the levels of cleaved caspase-8/7/9 in cells with DUXAP8 overexpression (Figure 3d**; Figure S3, bottom panel**). These findings indicate that the overexpression of DUXAP8 enhances the radioresistance of BC cells through activating PI3K/AKT/mTOR pathway and suppressing apoptosis.

### DUXAP8 acts as an upstream activator of EZH2 in BC cells

**Figure 4.**
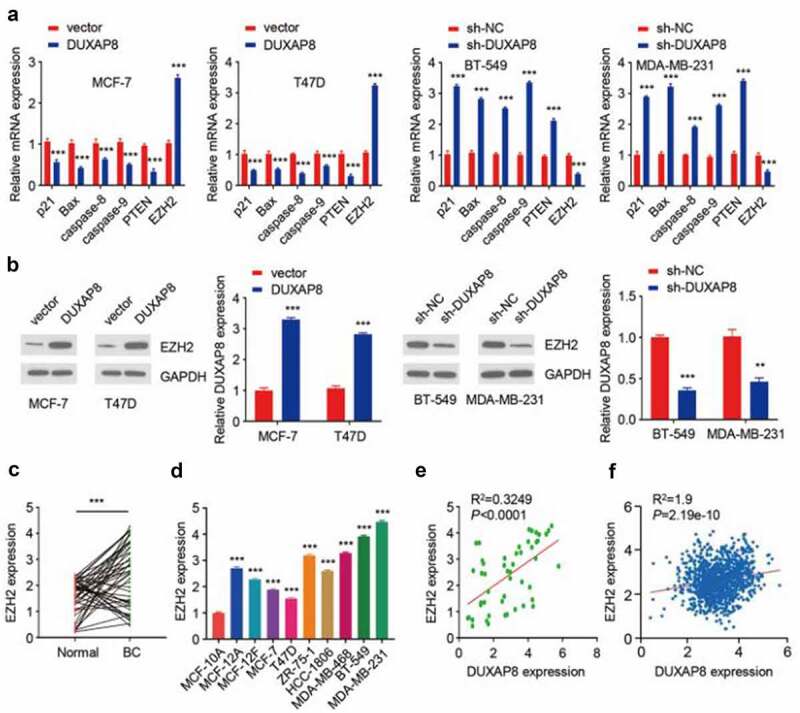
DUXAP8 acts as an upstream activator of EZH2 in BC cells.

To get more insight into the mechanism by which DUXAP8 promotes the radiosensitivity of BC cells, we examined the impact of DUXAP8 overexpression or DUXAP8 knockdown on genes involved in cell cycle regulation and apoptosis using qRT-PCR. The overexpression of DUXAP8 significantly decreased the expression of p21, Bax, caspase-8, caspase-9, PTEN, but increased the mRNA level of EZH2 in MCF-7 and T47D cells (Figure 4a). In contrast, the downregulation of DUXAP8 showed the opposite effects in BT-549 and MDA-MB-231 cells (Figure 4a). The effects of DUXAP8 overexpression and DUXAP8 knockdown on EZH2 protein levels were confirmed by Western blot (Figure 4b). We also determined the expression level of EZH2 in BC tissues and the adjacent normal tissues, and the results showed that there was an upregulation of EZH2 in BC tissues (Figure 4c). The mRNA levels of EZH2 were also significantly higher in BC cells than that of the normal breast epithelial MCF-10A cells (Figure 4d). Further, correlation analysis revealed that there was a positive correlation between DUXAP8 and EZH2 mRNA expression in the BC tissues (Figure 4e). A similar positive correlation between DUXAP8 and EZH2 expression was observed in the TCGA BC dataset using the ENCORI database (http://starbase.sysu.edu.cn) (figure 4f). These data suggest that DUXAP8 acts as an upstream activator of EZH2 in BC cells.

(**a**) qRT-PCR assays of p21, Bax, caspase-8, caspase-9, PTEN, and EZH2 expression in MCF-7 and T47D cells after the overexpression of DUXAP8, and in BT-549 and MDA-MB-231 cells after DUXAP8 knockdown. (**b**) Western blotting analysis of EZH2 level in MCF-7 and T47D cells with the overexpression of DUXAP8, and in BT-549 and MDA-MB-231 cells with DUXAP8 knockdown. (**c**) qRT-PCR assays of EZH2 levels in BC tissues and adjacent normal tissues. (**d**) qRT-PCR assays of EZH2 levels in BC cells and in MCF-10A cells. (**e**) Correlation analysis revealed that there was a significant positive correlation between DUXAP8 level and EZH2 mRNA expression in 50 BC tissues. (**f**) The association between DUXAP8 and EZH2 expression in the TCGA BC dataset was analyzed using the ENCORI database. *P < .05; ***P < .001.

### DUXAP8 serves as a scaffold for EZH2 to epigenetically silence EZH2 target genes

**Figure 5.**
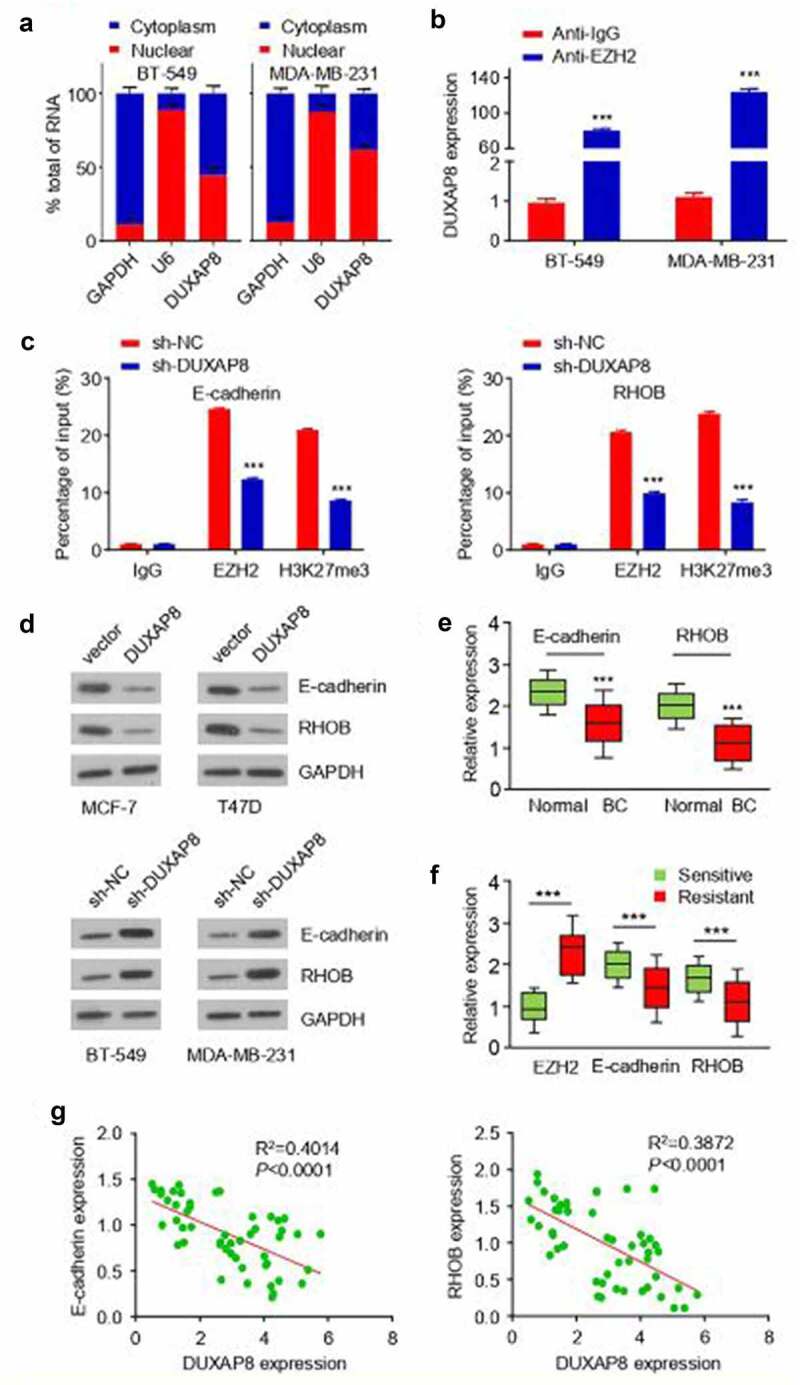
DUXAP8 serves as a molecular scaffold for EZH2 to epigenetically silence EZH2 target genes.

Then we prepared the nuclear and cytoplasmic fractions in BT-549 and MDA-MB-231 cells, and qRT-PCR results showed that DUXAP8 was predominantly localized in the nuclear fraction (Figure 5a). Using an online algorithm, RPISeq (http://pridb.gdcb.iastate.edu/RPISeq/), we found a possibility that DUXAP8 might directly target EZH2 protein since the DUXAP8-EZH2 interaction pair had a high score (interaction probability: RF = 0.75; SVM = 0.98). To investigate the potential interaction between DUXAP8 and EZH2 in BT-549 and MDA-MB-231 cells, RIP assays were performed. We found that DUXAP8 was significantly enriched with the EZH2 antibody when compared to the IgG control (Figure 5b). We therefore hypothesized that that DUXAP8 could bind to EZH2 to modulate its transcriptional regulation activity in BC cells.

(**a**) RNA was extracted from the nuclear and cytosolic fractions of BT-549 and MDA-MB-231 cells. qRT-PCR assay was performed to measure DUXAP8 level in the nuclear and cytoplasmic fractions. GAPDH was used as a cytoplasmic marker, and U6 was used as a nuclear marker. (**b**) RIP assays showed the association between DUXAP8 and EZH2 in BT-549 and MDA-MB-231 cells. (**c**) ChIP-qPCR assays showed that DUXAP8 is required for the binding of EZH2 to the promoter regions of *E-cadherin* (left) and *RHOB* (right) in MDA-MB-231 cells, as well as to reduce the level of H3K27me3. (**d**) Western blot analysis of E-cadherin and RHOB level in BT-549 and MDA-MB-231 cells following DUXAP8 knockdown, and in MCF-7 and T47D cells overexpressing DUXAP8. (**e**) qRT-PCR assays of E-cadherin and RHOB expression in BC tissues compared with adjacent normal tissues. (**f**) qRT-PCR assays of E-cadherin, RHOB and EZH2 expression in radioresistant BC tissues and radiosensitive BC tissues. (**g**) Correlation analysis showed negative correlations between the expression of DUXAP8 and E-cadherin/RHOB in BC tissues. ***P < .001.

E-cadherin and RHOB are known as EZH2 target genes ^1322^, which can modulate the radioresistance in BC cells ^2324^. To investigate whether DUXAP8 regulates the expression of E-cadherin and RHOB by affecting the bindings of EZH2 to the target gene promoter, we carried out ChIP-qPCR assays using anti-EZH2 and H3K27me3 antibody in MDA-MB-231 cells with DUXAP8 knockdown. Our results showed that the knockdown of DUXAP8 not only decreased the binding ability of EZH2 to the promoter, but also attenuated the level of repressive epigenetic marker H3K27me3 at promoter regions of E-cadherin and RHOB in MDA-MB-231 cells (Figure 5c). On the contrary, the protein levels of E-cadherin and RHOB in BT-549 and MDA-MB-231 were downregulated following overexpression of DUXAP8, but showed upregulation upon DUXAP8 knockdown (Figure 5d). Consistently, qRT-PCR assays showed that E-cadherin and RHOB expression was significantly downregulated in BC tissues (with a higher DUXAP level) compared with adjacent normal tissues (Figure 5e). Furthermore, we found that there was a significant downregulation of E-cadherin and RHOB, and an upregulation of EZH2 in radioresistant BC tissues compared with radiosensitive BC tissues (figure 5f). Pearson’s correlation analysis further revealed the negative correlations between DUXAP8 and E-cadherin or RHOB in BC tissues (Figure 5g). Taken together, these data suggest that DUXAP8 functions as a scaffold lncRNA to facilitate the targeting of EZH2 to the promoter regions of E-cadherin and RHOB and their epigenetic silencing.

### DUXAP8 promotes radioresistance of BC cells by regulating the EZH2-E-cadherin/RHOB axis

**Figure 6.**
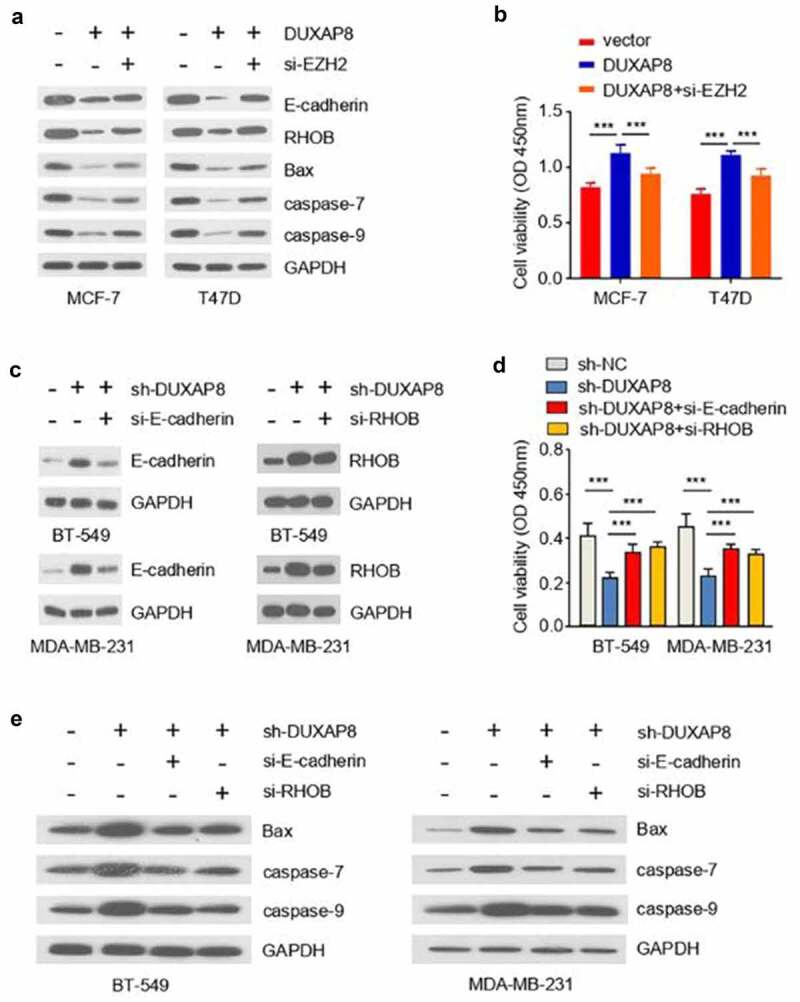
DUXAP8 promotes radioresistance of BC cells by regulating the EZH2-E-cadherin/RHOB axis.

We next sought to explore whether DUXAP8 regulates the radioresistance of BC cells by regulating the EZH2-E-cadherin/RHOB axis. To this end, we transfected MCF-7 and T47D cells overexpressing DUXAP8 with EZH2 siRNA to silence EZH2, and the cells were irradiated with a dose of 8 Gy X-radiation. Using qRT-PCR and Western blot analysis, we showed that the expression of E-cadherin and RHOB was deceased by DUXAP8 overexpression, which was partially restored by EZH2 silencing (Figure 6a). This was also accompanied by the increase of caspase-7/9 and Bax upon EZH2 silencing. Furthermore, CCK-8 assays demonstrated that the promoted cell survival by DUXAP8 overexpression was largely impaired upon silencing EZH2 in MCF-7 and T47D cells (Figure 6b). These results suggest that DUXAP8 promotes the radioresistance of BC cells by upregulating EZH2.

(**a**) MCF-7 and T47D cells overexpressing DUXAP8 were transfected with EZH2 siRNA, and irradiated with a dose of 8 Gy X-radiation. Western blot analyses of the indicated proteins were shown. (**b**) CCK-8 viability assay demonstrated that silencing EZH2 attenuated the effect of DUXAP8 overexpression on cell survival in MCF-7 and T47D cells upon irradiation. (**c**) BT-549 and MDA-MB-231 cells with (or without) DUXAP8 knockdown were transfected with E-cadherin (or RHOB)-siRNA, and these cells were irradiated with a dose of 8 Gy X-radiation. Western blot analysis showed that E-cadherin and RHOB levels induced by DUXAP8 knockdown were reduced by transfection with E-cadherin or RHOB) siRNA respectively in BT-549 and MDA-MB-231 cells. (**d**) CCK-8 viability assay showed that cell survival suppressed by DUXAP8 knockdown after irradiation was promoted by the silencing of E-cadherin or RHOB in BT-549 and MDA-MB-231 cells. (**e**) Western blot analysis showed that the increased expression of Bax and cleaved caspase-7/9 caused by DUXAP8 knockdown after irradiation was reduced by the silencing of E-cadherin or RHOB in BT-549 and MDA-MB-231 cells. ***P < .001.

Next, we transfected BT-549 and MDA-MB-231 cells with DUXAP8 knockdown with E-cadherin or RHOB-siRNA, and the cells were subjected to a dose of 8 Gy X-radiation. The expression of E-cadherin or RHOB was increased by DUXAP8 knockdown, but was reduced by the transfection with E-cadherin or RHOB-siRNA in BT-549 and MDA-MB-231 cells (Figure 6c). We noted that the suppression on cell survival by DUXAP8 knockdown was partially alleviated upon the silencing of E-cadherin or RHOB in BT-549 and MDA-MB-231 cells (Figure 6d). The rescued cell survival phenotype upon the silencing of E-cadherin or RHOB was accompanied by the downregulation of Bax and cleaved caspase-7/9 in BT-549 and MDA-MB-231 transfected with E-cadherin or RHOB-siRNA (Figure 6e). These data suggest that DUXAP8 regulates the radioresistance of BC cells by targeting the EZH2-E-cadherin/RHOB axis.

### *DUXAP8 promotes radioresistance of BC cells* in vivo

**Figure 7.**
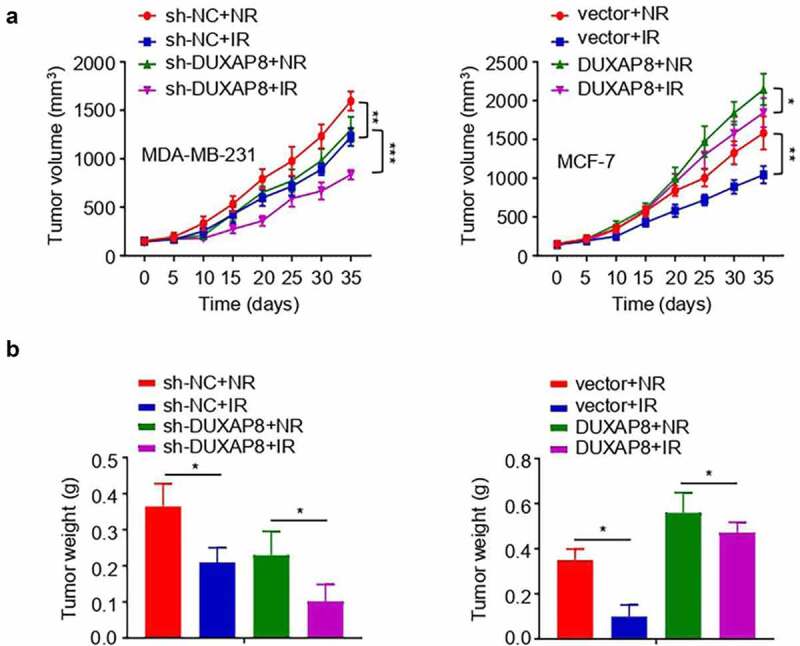
DUXAP8 promotes radioresistance of BC cells *in vivo.*

To evaluate the effects of DUXAP8 on radiosensitivity *in vivo*, MDA-MB-231 cells with or without DUXAP8 knockdown, and MCF-7 cells with or without DUXAP8 overexpression, were injected into the nude mice to establish the xenograft tumorigenesis model. The knockdown of DUXAP8 sensitized MDA-MB-231 cells to irradiation *in vivo*, as evidenced by the reduced tumor volume and weight in DUXAP8 knockdown group (Figure 7 a and b). In contrast, the overexpression of DUXAP8 increased the radioresistance of BC cells *in vivo* (Figure 7 a and b). Collectively, these results suggest that a high level of DUXAP8 expression contributes to the radioresistance of BC in the xenograft model.

(**a, b**) MDA-MB-231 cells with or without DUXAP8 knockdown, or MCF-7 cells with or without DUXAP8 overexpression were used to establish BC xenograft models. (**a**) Tumor volume was recorded in different groups with or without irradiation. (**b**) The weights of the tumors in different groups with or without irradiation were measured after the sacrifice of the mice. *P < .05; ***P < .01; ***P < .001.

## Discussion

Recently, lncRNAs have been identified as critical regulators of radiosensitivity in several types of cancer, including BC.^13^ However, little is known about the role of lncRNA DUXAP8 in regulating the radioresistance of BC cells. In this study, we provided convincing evidence that DUXAP8 expression is increased in radioresistant BC tissues, and the upregulation of DUXAP8 dramatically enhances the radioresistance of BC cells in both *in vitro* and *in vivo* models. These data suggest that DUXAP8 overexpression confer survival advantages under irradiation, and the development of the therapeutic strategies targeting DUXAP8 may offer new opportunities to overcome the resistance of BC cells to radiation.

DUXAP8 is frequently overexpressed in human tumors ^12131415161718^, and the upregulation of DUXAP8 is associated with more aggressive phenotypes of different cancers.^21–23^ Increased DUXAP8 expression was reported to be associated with larger tumor size, and advanced pathological stage of pancreatic cancer.^23^ Furthermore, a high level of DUXAP8 expression was associated with a poorer prognosis in many cancers.^21–28^ In agreement with these findings, our data validated that the overexpression of DUXAP8 predicted worse survival in patients with BC, indicating that DUXAP8 may serve as a biomarker for unfavorable prognosis BC patients.

The oncogenic roles of DUXAP8 have been reported in gastric cancer,^12^ lung cancer,^13^ bladder cancer,^14^ renal cell carcinoma,^15^ hepatocellular carcinoma,^16^ neuroblastoma,^17^ and colorectal cancer.^18^ DUXAP8 can promote cell proliferation, migration, invasion, and metastasis ^12131415161718^. In addition, the knockdown of DUXAP8 significantly represses epithelial-mesenchymal transition (EMT) in lung cancer and hepatocellular carcinoma.^27,28^ However, the biological functions of DUXAP8 in BC have not been elucidated. Our gain-of-function and loss-of-function experiments revealed the critical role of DUXAP8 in enhancing the radioresistance of BC cells. However, the mechanisms underlying the upregulation of DUXAP8 in BC remain to be determined.

LncRNAs are implicated in the regulation of tumor cell radioresistance via different mechanisms, including DNA damage repair, cell cycle arrest, apoptosis, autophagy, EMT, and cancer stemness ^58^. In our study, we provided evidence that DUXAP8 is capable of modulating DNA damage levels and radiation-induced apoptosis in BC cells. Furthermore, EZH2 has been shown to silence the expression of E-cadherin and RHOB through histone H3K27 trimethylation ^132229^. The overexpression of E-cadherin and RHOB were reported to sensitize BC cells to radiotherapy ^2324^. Consistent with these previous findings, we have demonstrated that DUXAP8 could promote radioresistance by epigenetically repressing the expression of E-cadherin and RHOB through targeting with EZH2. EZH2 can also regulate EMT by repressing the expression of E-cadherin,^29^ and the downregulation of EZH2 expression reduced the stemness of cancer cells in BC.^30^ Further studies are required to explore the possibility that DUXAP8 affects the EMT process and cancer stem cell properties in an EZH2-dependent manner, thereby leading to the development of radioresistance in BC.

It has been reported that lncRNAs can interplay with miRNAs and antagonize the repressive activity of miRNAs on gene expression in tumor cells.^5^ For instance, DUXAP8 was shown to function as a sponge for miR-577 to promote the migration and invasion of colorectal cancer cells.^18^ Moreover, DUXAP8 enhances the progression of renal cell carcinoma via downregulating miR-126.^15^ Interestingly, miR-126 has been shown to increase chemosensitivity in drug-resistant gastric cancer cells by targeting EZH2.^31^ We found that forced expression of DUXAP8 induced the levels of EZH2 in BC cells. Whether DUXAP8 regulates EZH2 expression through intermediate miRNAs remain to be further explored.

Abnormal EZH2 expression and the constitutive activation of PI3K/AKT/mTOR signaling contribute to the malignant progression of BC.^31–33^ In this study, we found that DUXAP8 could enhance the radioresistance in BC cells via recruiting EZH2 to the target genes and by activating the PI3K/AKT/mTOR pathway. Interestingly, a previous study showed that lncRNA UCA1 promotes gastric cancer cell proliferation by inducing EZH2 expression and activating AKT signaling.^34^ EZH2 and AKT signaling could also regulate the expression of each other in gastric cancer cells,^34^ and the treatment with EZH2 inhibitor (EZP005687) could decrease the phosphorylation and activity of AKT in gastric cancer cells.^34^ On the other hand, the administration of a specific PI3K inhibitor (LY49002) resulted in a significant reduction in EZH2 expression.^34^ Based on these results, it is likely that EZH2 and PI3K/AKT/mTOR signaling might constitute a positive feedback loop to reinforce the radioresistance in BC cells upon DUXAP8 overexpression. However, the relationship between EZH2 and PI3K/AKT/mTOR pathway, as well as the potential impact of their interplay on the aggressiveness and radioresistance of BC cells warrant further studies.

## Conclusions

In conclusion, our study uncovered a novel role of lncRNA DUXAP8 in augmenting the radioresistance in BC by activating PI3K/AKT/mTOR pathway and repressing E-cadherin and RHOB expression through targeting EZH2. These findings suggest that DUXAP8 represents a promising therapeutic target for the clinical management of radiosensitivity in BC patients.

## Supplementary Material

Supplemental Material

## Data Availability

The datasets used and/or analyzed during the current study are available from the corresponding author via email request.

## References

[1] Ferlay J, et al. Cancer incidence and mortality worldwide: sources, methods and major patterns in GLOBOCAN 2012. Int J Cancer. 2015;136:E359–E386. doi:10.1002/ijc.29210. 25220842

[2] Youlden DR, Cramb SM, Dunn NAM, Muller JM, Pyke CM, Baade PD. The descriptive epidemiology of female breast cancer: an international comparison of screening, incidence, survival and mortality. Cancer Epidemiol. 2012;36:237–248. doi:10.1016/j.canep.2012.02.007. 22459198

[3] Jagsi R. Progress and controversies: radiation therapy for invasive breast cancer. CA Cancer J Clin. 2014;64:135–152. doi:10.3322/caac.21209. 24357525

[4] Jameel JKA, Rao VSR, Cawkwell L, Drew PJ. Radioresistance in carcinoma of the breast. Breast. 2004;13:452–460. doi:10.1016/j.breast.2004.08.004. 15563851

[5] Dong P, Xiong Y, Yue J, Hanley SJB, Kobayashi N, Todo Y, Watari H. Long non-coding RNA NEAT1: a novel target for diagnosis and therapy in human tumors. Front. Genet. 2018;9(471). doi:10.3389/fgene.2018.00471. PMC6196292 30374364

[6] Bartel DP. MicroRNAs: genomics, biogenesis, mechanism, and function. Cell. 2004;116:281–297. doi:10.1016/S0092-8674(04)00045-5. 14744438

[7] Xu D, Dong P, Xiong Y, Yue J, Konno Y, Ihira K, Kobayashi N, Todo Y, Watari H. MicroRNA-361-mediated inhibition of HSP90 expression and EMT in cervical cancer is counteracted by oncogenic lncRNA NEAT1. Cells. 2020;9(632). doi:10.3390/cells9030632. PMC7140536 32151082

[8] Zhu J, et al. Molecular mechanisms of lncRNAs in regulating cancer cell radiosensitivity. Biosci Rep. 2019;39(BSR20190590):10.1042/BSR20190590 PMC6712435 31391206

[9] Wang B, et al. Long noncoding RNA LINC02582 acts downstream of miR-200c to promote radioresistance through CHK1 in breast cancer cells. Cell Death Dis. 2019;10(764):10.1038/s41419-019-1996-0 PMC6787210 31601781

[10] Cai Q, Jin L, Wang S, Zhou D, Wang J, Tang Z, Quan Z. Long non-coding RNA UCA1 promotes gallbladder cancer progression by epigenetically repressing p21 and E-cadherin expression. Oncotarget. 2017;8(29):47957–47968. doi:10.18632/oncotarget.18204. 28624787 PMC5564618

[11] Paplomata E, The OR. PI3K/AKT/mTOR pathway in breast cancer: targets, trials and biomarkers. Ther. Adv. Med. Oncol. 2014;6(4):154–166. doi:10.1177/1758834014530023. 25057302 PMC4107712

[12] Ma HW, Xie M, Sun M, Chen T-Y, Jin -R-R, Ma T-S, Chen Q-N, Zhang E-B, He X-Z, De W, et al. The pseudogene derived long noncoding RNA DUXAP8 promotes gastric cancer cell proliferation and migration via epigenetically silencing PLEKHO1 expression. Oncotarget. 2016;8:52211–52224. doi:10.18632/oncotarget.11075. 28881724 PMC5581023

[13] Sun M, Nie F-Q, Zang C, Wang Y, Hou J, Wei C, Li W, He X, Lu K-H. The pseudogene DUXAP8 promotes non-small-cell lung cancer cell proliferation and invasion by epigenetically silencing EGR1 and RHOB. Mol. Ther. 2017;25:739–751. doi:10.1016/j.ymthe.2016.12.018. 28131418 PMC5363203

[14] Lin MG, Hong YK, Zhang Y, Lin BB, He XJ. Mechanism of lncRNA DUXAP8 in promoting proliferation of bladder cancer cells by regulating PTEN. Eur Rev Med Pharmacol Sci. 2021;22:3370–3377. 10.26355/eurrev_201806_15158 29917188

[15] Huang T, Wang X, Yang X, Ji J, Wang Q, Yue X, Dong Z. Long non-coding RNA DUXAP8 enhances renal cell carcinoma progression via downregulating miR-126. Med. Sci. Monit. 2018;24:7340–7347. doi:10.12659/MSM.910054. 30317248 PMC6198709

[16] Jiang H, Shi X, Ye G, Xu Y, Xu J, Lu J, Lu W. Up-regulated long non-coding RNA DUXAP8 promotes cell growth through repressing krüppel-like factor 2 expression in human hepatocellular carcinoma. Onco. Targets. Ther. 2019;12:7429–7436. doi:10.2147/OTT.S214336. 31571902 PMC6750713

[17] Nie L, Li C, Zhao T, Wang Y, Liu J. LncRNA double homeobox A pseudogene 8 (DUXAP8) facilitates the progression of neuroblastoma and activates Wnt/β-catenin pathway via microRNA-29/nucleolar protein 4 like (NOL4L) axis. Brain Res. 2020;1746(146947):146947. doi:10.1016/j.brainres.2020.146947. 32522628

[18] Du C, Wang HX, Chen P, Chen CH. STAT3-induced upregulation of lncRNA DUXAP8 functions as ceRNA for miR-577 to promote the migration and invasion in colorectal cancer through the regulation of RAB14. Eur Rev Med Pharmacol Sci. 2019;23:6105–6118. doi:10.26355/eurrev_201907_18424. 31364111

[19] Li S, Wu D, Jia H, Zhang Z. Long non-coding RNA LRRC75A-AS1 facilitates triple negative breast cancer cell proliferation and invasion via functioning as a ceRNA to modulate BAALC. Cell Death Dis. 2020;11(8):643. 32811810 10.1038/s41419-020-02821-2 PMC7434919

[20] Shi L, Hong X, Ba L, He X, Xiong Y, Ding Q, Yang S, Peng G. Long non-coding RNA ZNFX1-AS1 promotes the tumor progression and metastasis of colorectal cancer by acting as a competing endogenous RNA of miR-144 to regulate EZH2 expression. Cell Death Dis. 2019;10(150). doi:10.1038/s41419-019-1332-8. PMC6377660 30770796

[21] Chen Q, Cai J, Wang Q, Wang Y, Liu M, Yang J, Zhou J, Kang C, Li M, Jiang C, et al. Long noncoding RNA NEAT1, regulated by the EGFR pathway, contributes to glioblastoma progression through the WNT/ β -Catenin pathway by scaffolding EZH2. Clin. Cancer Res. 2018;24:684–695. doi:10.1158/1078-0432.CCR-17-0605. 29138341

[22] Xu L-J, Yu X-J, Wei B, Hui H-X, Sun Y, Dai J, Chen X-F. Long non-coding RNA DUXAP8 regulates proliferation and invasion of esophageal squamous cell cancer. European Review for Medical and Pharmacological Sciences. 2018;22(9):2646–2652. doi:10.26355/eurrev_201805_14959. 29771416

[23] Lian Y, Yang J, Lian Y, Xiao C, Hu X, Xu H. DUXAP8, a pseudogene derived lncRNA, promotes growth of pancreatic carcinoma cells by epigenetically silencing CDKN1A and KLF2. Cancer Commun. 2018;38:64. doi:10.1186/s40880-018-0333-9. PMC6235391 30367681

[24] Cao Q, Yu J, Dhanasekaran SM, Kim JH, Mani R-S, Tomlins SA, Mehra R, Laxman B, Cao X, Yu J, et al. Repression of E-cadherin by the polycomb group protein EZH2 in cancer. Oncogene. 2008;27:7274–7284. doi:10.1038/onc.2008.333. 18806826 PMC2690514

[25] Theys J, Jutten B, Habets R, Paesmans K, Groot AJ, Lambin P, Wouters BG, Lammering G, Vooijs M. E-Cadherin loss associated with EMT promotes radioresistance in human tumor cells. Radiother. Oncol. 2011;99:392–397. doi:10.1016/j.radonc.2011.05.044. 21680037 PMC4948667

[26] Srougi MC, Burridge K. The nuclear guanine nucleotide exchange factors Ect2 and Net1 regulate RhoB-mediated cell death after DNA damage. PLoS One. 2011;6:e17108–e17108. doi:10.1371/journal.pone.0017108. 21373644 PMC3044157

[27] Ji X, Tao R, Sun LY, Xu XL, Ling W. Down-regulation of long non-coding RNA DUXAP8 suppresses proliferation, metastasis and EMT by modulating miR-498 through TRIM44-mediated AKT/mTOR pathway in non-small-cell lung cancer. Eur Rev Med Pharmacol Sci. 2020;24:3152–3165. doi:10.26355/eurrev_202003_20682. 32271433

[28] Wei F, Yang L, Jiang D, Pan M, Tang G, Huang M, Zhang J. Long noncoding RNA DUXAP8 contributes to the progression of hepatocellular carcinoma via regulating miR-422a/PDK2 axis. Cancer Med. 2020;9:2480–2490. doi:10.1002/cam4.2861. 32022476 PMC7131864

[29] Luo HN, Jiang Y, Ma S, Chang H, Yi C, Cao H, Gao Y, Guo H, Hou J, Yan J, et al. EZH2 promotes invasion and metastasis of laryngeal squamous cells carcinoma via epithelial-mesenchymal transition through H3K27me3. Biochem. Biophys. Res. Commun. 2016;479:253–259. doi:10.1016/j.bbrc.2016.09.055. 27638307

[30] van Vlerken LE, Kiefer CM, Morehouse C, Li Y, Groves C, Wilson SD, Yao Y, Hollingsworth RE, Hurt EM, et al. EZH2 is required for breast and pancreatic cancer stem cell maintenance and can be used as a functional cancer stem cell reporter. Stem Cells Transl Med. 2013;2:43–52. doi:10.5966/sctm.2012-0036. 23283488 PMC3659740

[31] Wang P, Li Z, Liu H, Zhou D, Fu A, Zhang E. MicroRNA-126 increases chemosensitivity in drug-resistant gastric cancer cells by targeting EZH2. Biochem. Biophys. Res. Commun. 2016;479:91–96. doi:10.1016/j.bbrc.2016.09.040. 27622325

[32] Johnson J, Chow Z, Napier D, Lee E, Weiss HL, Evers BM, Rychahou P. Targeting PI3K and AMPKα signaling alone or in combination to enhance radiosensitivity of triple negative breast cancer. Cells. 2020;9(1253). doi:10.3390/cells9051253. PMC7291172 32438621

[33] Duan R, Du W, Guo W. EZH2: a novel target for cancer treatment. J Hematol Oncol. 2020;13(104). doi:10.1186/s13045-020-00937-8. PMC7385862 32723346

[34] Wang ZQ, Cai Q, Hu L, He C-Y, Li J-F, Quan Z-W, Liu B-Y, Li C, Zhu Z-G. Long noncoding RNA UCA1 induced by SP1 promotes cell proliferation via recruiting EZH2 and activating AKT pathway in gastric cancer. Cell Death Dis. 2017;8:e2839–e2839. doi:10.1038/cddis.2017.143. 28569779 PMC5520878

