# Supplementary material for: LncRNA DUXAP8 induces breast cancer radioresistance by modulating the PI3K/AKT/mTOR pathway and the EZH2-E-cadherin/RHOB pathway
Source: Cancer Biol Ther. 2022 Nov 3;23(1):1–13. doi: 10.1080/15384047.2022.2132008 (PMC9635553; doi:10.1080/15384047.2022.2132008)

## Cell Line Authentication Service

**STR Profiling Report**

**Sample From:** Shanghai GuanDao Biological Engineering Co., Ltd

**Sample Type:** Cell Line

**Testing Method:** STR Genotyping

**Report Time:** May 6, 2022

**Cell Line Authentication – STR Profiling Report**

### Sample code

Table 1. Sample Code

#### Customer’s code Company Code

MCF-7 20220428-25

**Sample Number**:1 **Sample Type**: Cell line **Testing Type**: STR **Testing Method**:

DNA was extracted by a commercial kit from CORNING (AP-EMN-BL-GDNA-250G). The twenty STRs including Amelogenin locus were amplified by six multiplex PCR and separated on ABI 3730XL Genetic Analyzer. The signals were then analyzed by the software GeneMapper.

### Data Interpretation:

Cell lines were authenticated using Short Tandem Repeat (STR) analysis asdescribed in 2012 in ANSI Standard (ASN-0002) by the ATCC Standards Development Organization (SDO) and in Capes-Davis et al., Match criteria for human cell line authentication: Where do we draw the line? Int J Cancer.2013;132(11):2510-9.

#### STR profile

**Test Results**

Table 2. STR and Amelogenin Genotyping Results of Cell line 20220428-25

| Loci | **Sample information** | | | **Cell Bank information** | | | |
| --- | --- | --- | --- | --- | --- | --- | --- |
|  | Sample | name：MCF-7 | | Cell line | name：MCF-7 | | |
|  | **Allele1** | | **Allele2 Allele3** | **Allele1** | | **Allele2** | **Allele3** |
| D5S818 | 11 | 12 | | 11 | 12 | | |
| D13S317 | 11 | 11 | | 11 | 11 | | |
| D7S820 | 8 | 9 | | 8 | 9 | | |
| D16S539 | 11 | 12 | | 11 | 12 | | |
| VWA | 14 | 15 | | 14 | 15 | | |
| TH01 | 6 | 6 | | 6 | 6 | | |
| AMEL | X | Y | | X | Y | | |
| TPOX | 9 | 12 | | 9 | 12 | | |
| CSF1PO | 10 | 10 | | 10 | 10 | | |
| D12S391 | 18 | 20 | |  | | | |
| FGA | 23 | 25 | |  | | | |
| D2S1338 | 21 | 23 | |  | | | |
| D21S11 | 30 | 30 | |  | | | |
| D18S51 | 14 | 14 | |  | | | |
| D8S1179 | 10 | 14 | |  | | | |
| D3S1358 | 16 | 16 | |  | | | |
| D6S1043 | 12 | 18 | |  | | | |
| PENTAE | 7 | 12 | |  | | | |
| D19S433 | 13 | 13 | |  | | | |
| PENTAD | 12 | 12 | |  | | | |
| D1S1656 | 11 | 15.3 | |  | | | |

#### database annotation

Figure 1. STR matching analysis


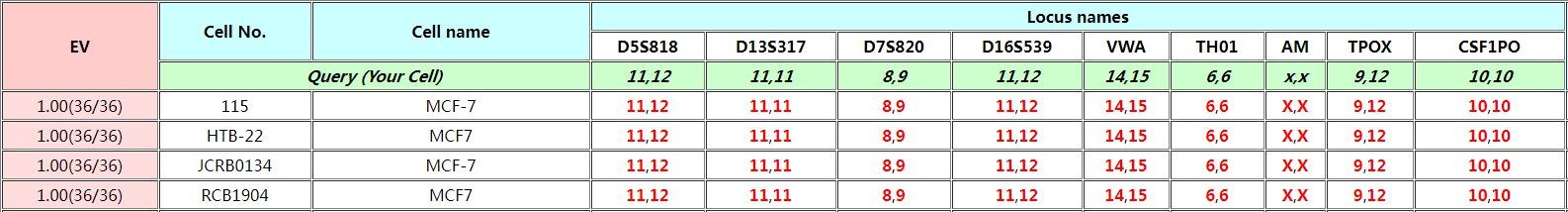


**Note:** The STR online match analysis of the test cell against DSMZ database, showing cell number (Cell No.) and cell name.

#### Authentication


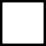
 The submitted sample profile is human, but not a match for any profile in the DSMZ STR database.


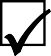
 The submitted profile is an exact match for the following human cell line(s) in the DSMZ


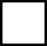
STR database (8 core loci plus Amelogenin): **MCF-7** .

The submitted profile is similar to the following DSMZ human cell line: /.

- **Note:** A cell line can be considered to be authenticated when 80% (exact match) of the alleles in its STR profile match profiles from tissue or other cell line samples from that donor or from database. Cell lines with between a 55% to 80% (similar) match require further profiling for investigation of relatedness.

# Appendix:

1. Genotyping Strategy and Site Distribution

Table S1. Experimental Strategy and Sites

|  | **Strategy 1** | **Strategy 2** | **Strategy 3** | **Strategy 4** |
| --- | --- | --- | --- | --- |
| 1 | AMEL | D16S539 | TH01 | D8S1179 |
| 2 | D3S1358 | D18S51 | VWA | D12S391 |
| 3 | D1S1656 | D2S1338 | D21S11 | D19S433 |
| 4 | D6S1043 | CSF1PO | D7S820 | FGA |
| 5 | D13S317 | PENTAD | D5S818 |  |
| 6 | PENTAE |  | TPOX |  |

*The allele match algorithm compares the 8 core loci plus amelogenin only, even though alleles from all lociwill be reported when available.*

1. DSMZ tools was used to carry on the cell line comparison, which contains 2455 cell lines STR data from ATCC, DSMZ, JCRB ,ECACC，GNE and RIKEN databases. If the cell is not included in the above cell library, users need to compared with other databases.

**Technician:** Xiaolu Li **Checked by:** Liqun Zhao **Issued by:** Xue Han **Issue date:** May 6, 2022


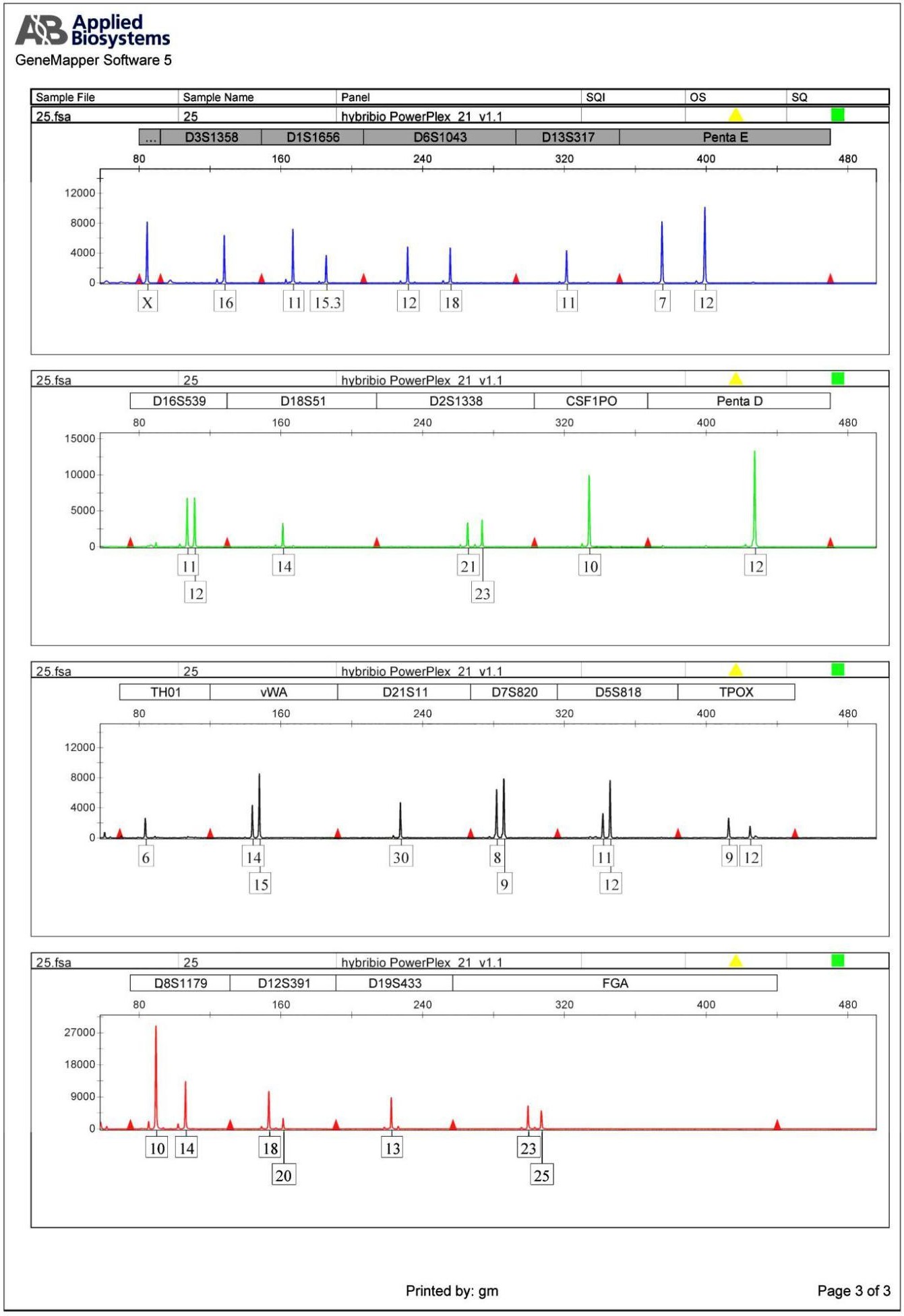

Supplement: Supplemental Material [file KCBT_A_2132008_SM6119.zip › MCF_7_STR_report_Final (1).docx]
