# Supplementary figures and images for: LncRNA DUXAP8 induces breast cancer radioresistance by modulating the PI3K/AKT/mTOR pathway and the EZH2-E-cadherin/RHOB pathway
Source: Cancer Biol Ther. 2022 Nov 3;23(1):1–13. doi: 10.1080/15384047.2022.2132008 (PMC9635553; doi:10.1080/15384047.2022.2132008)

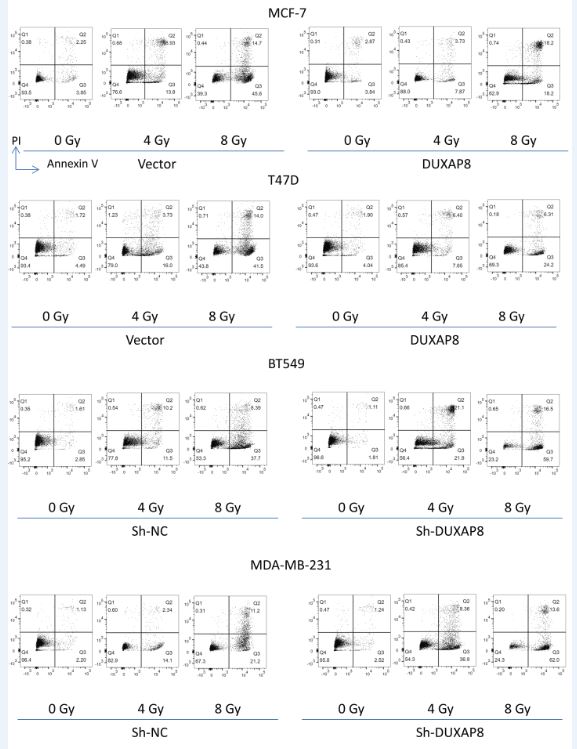

Supplement: Supplemental Material [file KCBT_A_2132008_SM6119.zip › Figure S1 (1).JPG]

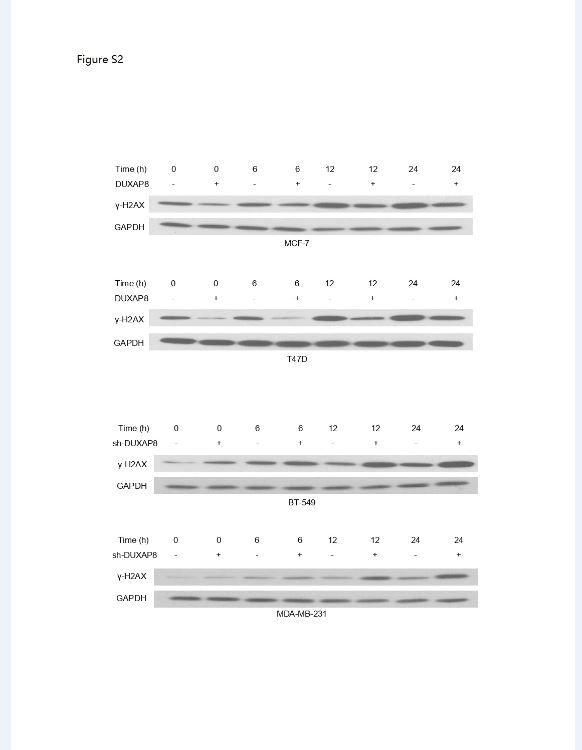

Supplement: Supplemental Material [file KCBT_A_2132008_SM6119.zip › Figure S2.JPG]

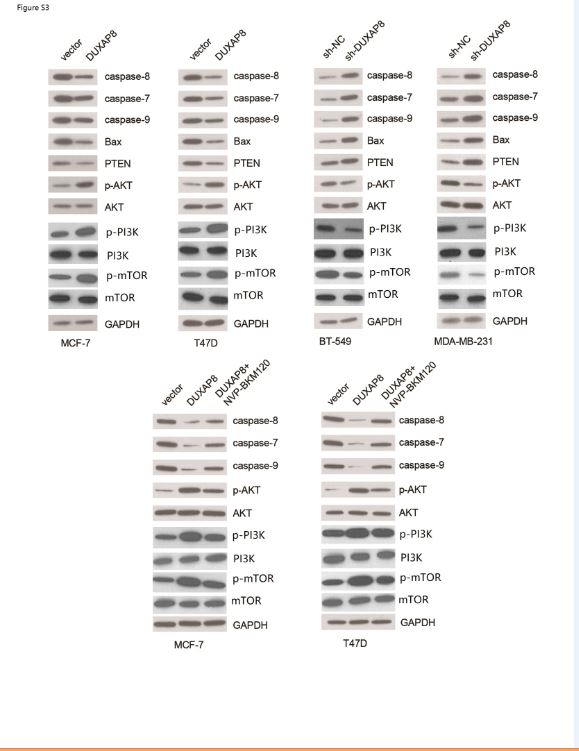

Supplement: Supplemental Material [file KCBT_A_2132008_SM6119.zip › Figure S3.JPG]

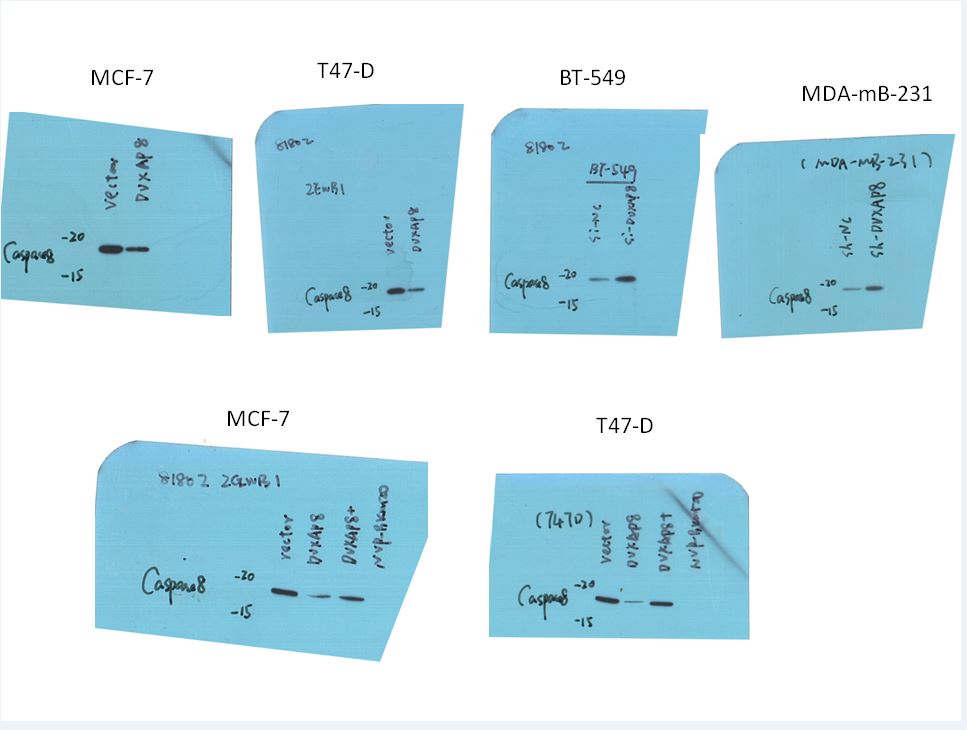

Supplement: Supplemental Material [file KCBT_A_2132008_SM6119.zip › Original WB blot of caspase 8.TIF]
